# Reduced HIF-1α Stability Induced by 6-Gingerol Inhibits Lung Cancer Growth through the Induction of Cell Death

**DOI:** 10.3390/molecules27072106

**Published:** 2022-03-24

**Authors:** Min Jeong Kim, Jin Mo Ku, Yu-Jeong Choi, Seo Yeon Lee, Se Hyang Hong, Hyo In Kim, Yong Cheol Shin, Seong-Gyu Ko

**Affiliations:** 1 Department of Science in Korean Medicine, Graduate School, Kyung Hee University, 26 Kyungheedae Rd., Dongdaemun-gu, Seoul 02447, Korea; jung8328@hanmail.net (M.J.K.); ehowlqk11@naver.com (Y.-J.C.); dltjdus0225@naver.com (S.Y.L.); hi9265@nate.com (H.I.K.); syc99@khu.ac.kr (Y.C.S.); 2 Institute of Safety and Effectiveness Evaluation for Korean Medicine, 26 Kyungheedae Rd., Dongdaemun-gu, Seoul 02447, Korea; saory_ykm@naver.com (J.M.K.); sehyang@khu.ac.kr (S.H.H.); 3 Department of Preventive Medicine, College of Korean Medicine, Kyung Hee University, 26 Kyungheedae Rd., Dongdaemun-gu, Seoul 02447, Korea

**Keywords:** 6-gingerol, cancer, HIF-1α, HSP90, metastasis

## Abstract

Lung cancer (LC) is the leading global cause of cancer-related death, and metastasis is a great challenge in LC therapy. Additionally, solid cancer, including lung, prostate, and colon cancer, are characterized by hypoxia. A low-oxygen state is facilitated by the oncogene pathway, which correlates with a poor cancer prognosis. Thus, we need to understand the related mechanisms in solid tumors to improve and develop new anticancer strategies. The experiments herein describe an anticancer mechanism in which heat shock protein 90 (HSP90) stabilizes HIF-1α, a master transcription factor of oxygen homeostasis that has been implicated in the survival, proliferation and malignant progression of cancers. We demonstrate the efficacy of 6-gingerol and the molecular mechanism by which 6-gingerol inhibits LC metastasis in different oxygen environments. Our results showed that cell proliferation was inhibited after 6-gingerol treatment. Additionally, HIF-1α, a transcriptional regulator, was found to be recruited to the hypoxia response element (HRE) of target genes to induce the transcription of a series of target genes, including MMP-9, vimentin and snail. Interestingly, we found that 6-gingerol treatment suppressed activation of the transcription factor HIF-1α by downregulating HSP90 under both normoxic and hypoxic conditions. Furthermore, an experiment in an in vivo xenograft model revealed decreased tumor growth after 6-gingerol treatment. Both in vitro and in vivo analyses showed the inhibition of metastasis through HIF-1α/HSP90 after 6-gingerol treatment. In summary, our study demonstrates that 6-gingerol suppresses proliferation and blocks the nuclear translocation of HIF-1α and activation of the EMT pathway. These data suggest that 6-gingerol is a candidate antimetastatic treatment for LC.

## 1. Introduction

Lung cancer (LC) is one of the leading causes of global health issues, as it is the most common malignant tumor in men and women worldwide [1,2]. Non-small-cell lung cancer (NSCLC) is the main subtype of LC, accounting for 85% of all LC cases [3]. Despite advances in various treatment options, the overall 5-year survival rate for stage NSCLC is 1–5%, and approximately 70% of patients are diagnosed at an advanced stage with local metastasis [4,5]. As the invasion and metastasis of cancer cells are key to patient survival, a better understanding of the molecular basis of cancer cell migration would provide insight into successful therapies [6,7]. Thus, we need to better understand the cellular origins and molecular pathways of each of these subtypes.

Recent studies revealed that interest in herbal medicines has played an important role in the prevention of many diseases, such as cancer, obesity, and neuroprotection, due to the low side effects and high stability of these medicines [8,9,10]. The anticancer efficacy of plant-derived natural compounds in LC has been demonstrated [11,12]. One herbal medicine, *Zingiber* *officinale Roscoe*, has been used medicinally throughout the world since antiquity. Ginger contains phenolic substances, such as gingerol, shogaol and paradol, which are collectively known to exhibit a variety of biological activities, including antioxidant, antitumor and anti-inflammatory activities [13,14].

6-Gingerol, 1-[4′-hydroxy-3′-methoxyphenyl]-5-hydroxy-3-decanone, one of the major active components of ginger, has been reported to carry out a variety of biochemical and pharmacological activities, including its anti-inflammatory, antioxidant, and antitumor activities [15,16,17,18]. However, whether 6-gingerol, a compound in ginger, can suppress metastasis in LC cell lines has not been reported. Consequently, we investigated the molecular mechanism of the effect of 6-gingerol on LC in vitro and its antitumor effect in vivo.

In recent studies, many solid tumors are often characterized by hypoxia microenvironment including breast, lung, prostate, and ovary, induce hypoxia-inducible factor (HIF-1α) overexpression in the tumors microenvironment which a crucial role of transcription factor and molecular alteration [19,20]. Under hypoxia condition, hypoxia has been reported to reactive processes related to tumor progression such as angiogenesis, metabolic reprogramming, invasion and metastasis [21].

Metastasis, the loss of adhesion between cells that acquire mesenchymal properties (MET), refers to the spread of tumor cells through the bloodstream in the lymphatic system to other organs in the body [22]. Metastatic tumor cells undergo epithelial-mesenchymal transition (EMT), which is a developmental program implicated in tumor progression and metastatic properties [23]. During EMT, epithelial cells lose their polarity, and this process is a vital mechanism for the acquisition of a malignant phenotype with invasion [24]. However, the mechanisms that induce EMT to promote invasion and metastasis have not been clarified in recent reports.

In LC patients, high levels of HIF-1α correlate with a poor prognosis, a malignant phenotype and chemo- and radiotherapeutic resistance [25]. Furthermore, HIF-1α overexpression indicates the high potential of hypoxic cancer cells to develop a metastatic phenotype [26]. Low oxygen availability, a condition known as hypoxia, leads to HIF-1α and HIF-2α protein stabilization, dimerization with HIF-1β, DNA binding and target gene transcription activation [27,28]. Additionally, the binding of heat shock protein 90 (HSP90), a molecular chaperone, to HIF-1α is required to stabilize HIF-1α prior to its dimerization with HIF-1β and the intracellular accumulation of HIF-1α, which leads to the expression of many gene-encoded proteins [29,30].

In the present study, we analyzed signal activation to explain the mechanism by which 6-gingerol affects LC cells in vitro and in a xenograft model. The results show that 6-gingerol is mainly involved in proliferation inhibition through the interaction between HIF-1α and the EMT pathway under hypoxic conditions. Therefore, new drugs for better LC treatment are needed.

## 2. Material and Method

### 2.1. Reagents

Dulbecco’s phosphate-buffered saline (DPBS) and Roswell Park Memorial Institute-1640 (RPMI-1640) medium were obtained from WELGENE (Gyeongsan, Korea). 6-Gingerol, DMSO, and MTT were purchased from Sigma (St. Louis, MO, USA).

### 2.2. Cell Culture Preparation of a CoCl_2_ Stock Solution and Hypoxia Treatment

Human LC cells (H460, A549, and H1299 cells) were obtained from the American Type Collection (ATCC). The cancer cell lines were maintained in RPMI-1640 medium supplemented with 10% fetal bovine serum (JR Scientific) and 100 U/mL antibiotic-antimycotics (Invitrogen, Carlsbad, CA, USA) at 37 °C in a humidified incubator with 5% ${\mathrm{CO}}_{2}$.

### 2.3. Preparation of a CoCl_2_ Stock Solution and Hypoxia Treatment

A stock solution of 3 mM cobalt chloride (${\mathrm{CoCl}}_{2}$) was prepared in distilled water and diluted in medium to obtain the final desired concentration (100 μM). Cells were seeded in culture plates. After 24 h, the cells were washed with PBS, and the cell medium was changed to medium containing ${\mathrm{CoCl}}_{2}$.

### 2.4. Cell Viability Assay

Cell viability was assessed using the MTT assay. Human LC cells (H460, A549, H1299) were plated in 96-well culture plates at a density of 5.0 × 10^3^ and incubated for 24 h. After 24 h, the cells were treated with 6-gingerol (0, 50, or 100 μM) for 24 h. After incubation, an MTT solution (0.5 mg/mL) was added to each well, and the plates were incubated in the dark at 37 °C for 1 h. The medium was removed, formazan was dissolved in DMSO, and the optical density at 570 nm was measured using an ELISA plate reader (Versa Max, Molecular Devices, Silicon Valley, CA, USA).

### 2.5. Analysis of Cell Cycle Arrest

Cell cycle arrest was analyzed with PI single staining and a flow cytometer. Cells were seeded in 60 mm cell culture plates at a density of 3.0 × 10^3^. After 24 h, 6-gingerol was added (0, 100 μM) and incubated for 24 h. The cells were harvested, washed twice with PBS and then fixed with 95% ethanol at 4 °C for 30 min. After fixation, the cells were incubated with 1 U/mL RNase A and 10 μg/mL PI for 30 min at room temperature (RT) in the dark. The cell cycle was analyzed by a BD FACSCalibur flow cytometer (BD Bioscience, San Jose, CA, USA) following the manufacturer’s instructions.

### 2.6. TUNEL (BrdU-Red) Assay

Cellular DNA fragmentation was assessed using a TUNEL assay from Abcam (ab66110) following the manufacturer’s instructions. In brief, cells were seeded on cover slips at a density of 3.0 × 10^3^. After 24 h, 6-gingerol was added (0, 100 μM) and incubated for 24 h. Then, the cells were labeled with Br-dUTP (DNA labeling solution) for 1 h at 37 °C and incubated with anti–BrdU-Red antibody for 30 min at RT. Images were acquired using confocal microscopy (Carl Zeiss, Oberkochen, Germany).

### 2.7. Colony Formation Assay

Cell proliferation was analyzed using a colony formation assay. The cells were seeded in 6-well plates at a density of 1000 cells per well. The cells were allowed to attach overnight and then treated with 6-gingerol (0, 50, or 100 μM) for 7 days. The cells were stained with a 1% crystal violet solution (Amersham, Solon, OH, USA).

### 2.8. Examination of Cell Morphology by Microscopy

Cell morphology was analyzed by using phase-contrast microscopy. The cells were seeded in 6-well plates at a density of 1.0 × 10^3^ cells per well. After 24 h, 6-gingerol was added (0, 100 μM) and incubated for 24 h. The differences in morphology were observed by phase-contrast miscopy, and images were captured.

### 2.9. Proteome Profiler Human Ubiquitin Array Assay

A total of 1.0 × 10^6^ cells were seeded in 100 mm cell culture plates and treated with 6-gingerol (0, 100 μM). The cells were washed in PBS and lysed with lysis buffer containing PI for 30 min on ice. The protein lysates were microcentrifuged at 14,000× *g* for 5 min, and the supernatant was transferred to a clean e-tube. Protein quantification was performed using a Bio-Rad Bradford protein assay kit (Bio-Rad, Hercules, CA, USA). Five hundred micrograms of each whole-protein sample was analyzed with a Proteome Profiler Human Ubiquitin Array assay kit from R&D Systems (ARY027) according to the manufacturer’s procedure.

### 2.10. Immunofluorescence (IF) Staining

Images showing subcellular localization were obtained using a confocal microscope. A total of 3.0 × 10^3^ cells were seeded on coverslips and treated with 6-gingerol. After 24 h, the cells were washed twice in PBS, fixed with 4% paraformaldehyde and blocked with 3% BSA. Then, the cells were probed with primary antibodies overnight at 4 °C. The next day, the cells were probed with Alexa Fluor-conjugated secondary antibody for 1 h and stained with DAPI for 10 min at RT. Images were acquired using confocal microscopy (Carl Zeiss, Oberkochen, Germany).

### 2.11. Western Blot Analysis

Cells were washed in PBS and lysed with RIPA buffer containing PMSP, DTT, and PI for 20 min on ice. The protein lysates were separated by centrifugation at 13,000 rpm for 20 min at 4 °C. The supernatant was transferred to a clean e-tube. Protein quantification was performed using a Bio-Rad Bradford protein assay kit (Bio-Rad, Hercules, CA, USA). Fifteen micrograms of protein was separated on 6–15% sodium dodecyl sulfate (SDS)-polyacrylamide gels by electrophoresis and transferred to nitrocellulose membranes (protran nitrocellulose membrane, Whatman, UK). Then, the membranes were blocked in PBS with 0.1% Tween-20 containing 1% BSA and 1.5% skim milk for 1 h. The membranes were incubated with primary antibodies at 4 °C overnight. Then, the membranes were incubated with HRP-conjugated secondary IgG antibodies (Calbiochem, San Diego, CA, USA) and probed using an enhanced chemiluminescence detection system (Amersham ECL kit, Amersham Pharmacia Biotech Inc., Piscataway, NJ, USA).

Antibodies against HIF-1α (#79233), HSP90 (#4877), vimentin (#5741), snail (#3879), and GAPDH (#5174) were obtained from Cell Signaling Technology (Danvers, MA, USA). Antibodies against MMP-9 (sc-21733) were purchased from Santa Cruz Biotechnology (Dallas, TX, USA).

### 2.12. Wound Healing Assay

The ability of H460 and H1299 cells to migrate was measured with a wound healing assay. Cells were seeded in 6-well plates at a density of 3.0 × 10^3^ cells per well. After 24 h, the cell medium was changed to serum-free medium for starvation, and a straight line was made with a 1 mL pipette tip to create a wound. Then, the cells were washed with PBS to remove debris, and 6-gingerol (0, 100 μM) in regular medium was added and incubated for 24 h. Then, we observed the wound healing area and captured images using phase-contrast miscopy.

### 2.13. RT–PCR

mRNA expression in LC cell lines (H460, A549, and H1299 cells) was analyzed by RT–PCR. The cells were seeded in 100 mm cell culture dishes at a density of 1.0 × 10^6^ cells per dish. After 24 h, the cells were treated with JI017 (0, 40, 80, 120, and 200 μg/mL) and washed with PBS. RNA was isolated using an easy-BLUE RNA extraction kit (iNtRON Biotech, Seongnom, Gyeonggi-do, Korea). Briefly, 1 mL of easy-BLUE solution was added to the plates; 200 μL of chloroform was added to the lysate, and the tubes were inverted 6–7 times. The lysate was centrifuged at 13,000 rpm for 10 min at 4 °C. An appropriate volume of aqueous phase was transferred into another e-tube, and 400 μL of isopropanol was added; the solution was mixed by inverting the tubes 6–7 times. The samples were centrifuged at 13,000 rpm for 10 min, and the supernatant was removed without disturbing the pellet. Then, 1 mL of 75% ethanol was added, and the solution was mixed by inverting the tubes 4–5 times. The mixture was centrifuged for 1 min at RT, and the supernatant was discarded without disturbing the pellet. The remaining RNA pellet was dried and dissolved in 20–50 μL of RNase-free water. The concentration of the isolated RNA was determined using a NanoDrop ND-1000 spectrophotometer (NanoDrop Technologies Inc., Wilmington, DE, USA). DNase was added to the samples. Two micrograms of total cellular RNA from each sample was reverse-transcribed using a cDNA synthesis kit (TaKaRa, Otsu, Shinga, Japan). PCR was performed in a 20 μL reaction mixture containing DNA template, 10 pM of the corresponding gene-specific primers, 10 × Taq buffer, 2.5 mM dNTP mixture, and 1 unit of Taq DNA polymerase (TaKaRa, Otsu, Shiga, Japan). PCR was performed using the specific primers listed in Table 1.

### 2.14. Animal Studies

All procedures in animal experiments were approved by Kyung Hee University Institutional Animal Care and Use Committee (KHSASP-20-250).

To examine the antitumor effects of 6-gingerol, human LC cells were injected into BALB/C nu/nu male mice. Five-week-old athymic BALB/C nu/nu male mice were purchased from Raonbio (Seoul, Korea). After 7 days of acclimation (constant RT with a 12 h light/12 h dark cycle and fed a standard rodent diet and water), H460 cells were harvested and injected subcutaneously (5 × 10^6^ cells in 100 µL, PBS:Matrigel = 1:1) into the right flank of the mice. When the xenografts reached a volume of 80–100 mm^3^, the animals were randomized into 2 groups (n = 5): the control group (saline) and the 6-gingerol treatment group (5 mg/kg), the animals in which were treated with 6-gingerol by oral administration every 2 days. Tumor sizes were measured every 2 days for changes in tumor growth, and tumor volumes were calculated with a standard formula: length × width^2^/2. On the 21st day, mice were sacrificed using carbon dioxide, followed by cervical dislocation, and the tumor tissue was isolated for further study, such as immunohistochemical (IHC) analyses.

### 2.15. IHC Analysis

To examine the protein expression of tumor-related genes in tumor section samples, each serial frozen section was dried at RT for 20 min. After hydration, the samples were fixed with 4% paraformaldehyde and washed at 4 °C for 5 min. Then, the cells were blocked with bovine serum albumin (3% BSA) and probed with primary antibodies (1:100~1:400) overnight at 4 °C. Proteins were identified using anti-HIF-1α (#79233), anti-MMP-9 (sc-21733), and anti-Ki67 (ab16667) antibodies. The next day, the sample was washed and probed with secondary antibody for 30 min at RT. Then, the slide was incubated with Vectastain ABC reagent (Vector Laboratories, Inc., Burlingame, CA, USA, sk-4100) for 30 min. Immune complexes were revealed via incubation with 3,3′-diaminobenzidine (DAB) at RT for 1 min based upon the targeted antigen. The sections were counterstained with hematoxylin and dehydrated on slides in 75%, 95% and 100% ethanol for 1 min each, after which the sections were cleared in xylene for 5 min. Finally, the sections were mounted using mounting medium. Images were acquired using microscopy.

### 2.16. Statistical Analysis

The results are expressed as the mean ± standard deviation (SD) or mean ± SEM. Statistical significance was determined using one-way analysis of variance followed by Tukey–Kramer multiple comparisons test to evaluate the differences (*p* < 0.05) between the groups. All experiments were performed at least three times. All statistical analyses were performed using Prism software (GraphPad Software Inc., La Jolla, CA, USA).

## 3. Results

### 3.1. 6-Gingerol Treatment Inhibits the Growth of Human LC Cells

**Figure 1:**
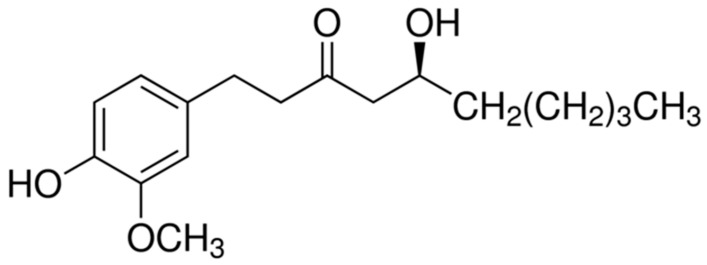
Chemical formula of 6-gingerol.

**Figure 2:**
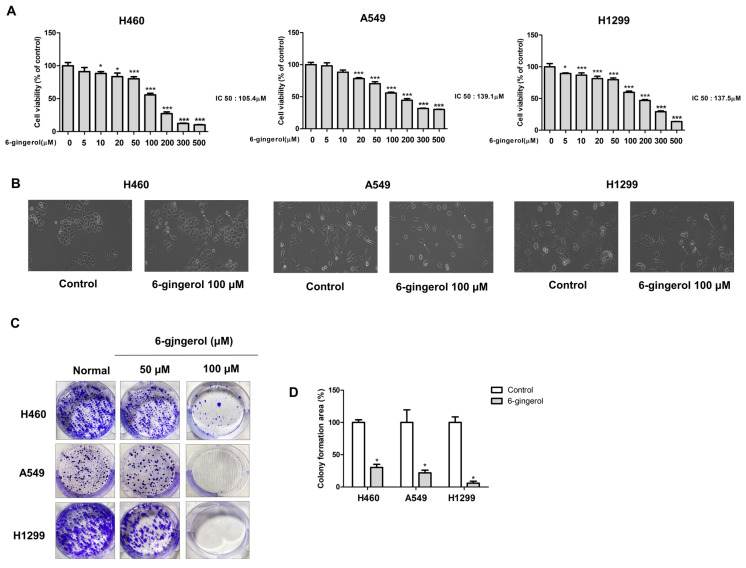
6-Gingerol treatment suppressed cell growth. (**A**) LC cell lines were treated with different concentrations of 6-gingerol for 24 h. Cell viability was measured by using the MTT assay. (**B**) H460, A549 and H1299 cells were treated with 6-gingerol for 24 h. Cell morphology was observed by phase-contrast microscopy at the original magnification. (**C**,**D**) H460, A549 and H1299 cells were exposed to 6-gingerol for 7 days. The effect on cell growth was assessed by using a colony formation assay. The data are presented as the mean ± SEM. * *p* < 0.05, and *** *p* < 0.001 compared to untreated cells.

We investigated whether 6-gingerol would affect the viability of LC cells (Figure 1). H460, A549, and H1299 cells were treated with various concentrations of 6-gingerol for 24 h. Then, cell viability was measured by the MTT assay. 6-Gingerol significantly inhibited the growth of H460, A549, and H1299 cells in a dose-dependent manner, with IC50 values for these cell lines of 105.4, 137.5, and 136.1 μM, respectively (Figure 2A). We observed numerous changes in the morphology of cells treated with 6-gingerol using a phase-contrast microscope. 6-Gingerol treatment induced morphological changes compared to the morphology of the control, and the LC cells were severely distorted and showed a reduction in cell volume and destabilization of the plasma membrane. The untreated cells displayed a normal, healthy shape with a distinct cytoskeleton (Figure 2B). Additionally, 6-gingerol treatment significantly reduced colony formation and the size of cell colonies in a dose-dependent manner compared with those in the control group (Figure 2C). These results suggest that 6-gingerol treatment has an inhibitory growth effect on human LC cell lines.

### 3.2. 6-Gingerol Treatment Suppressed the Proliferation of LC Cell Lines In Vitro

**Figure 3:**
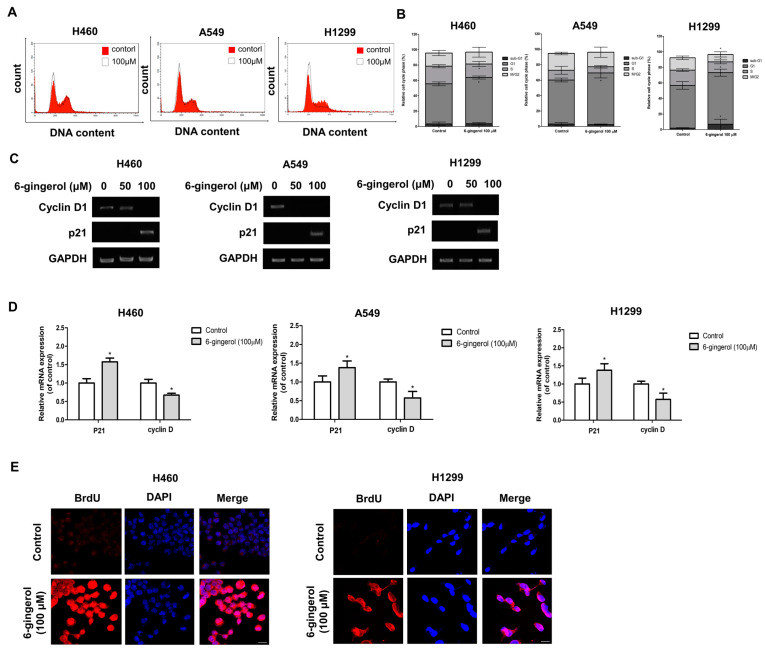
6-Gingerol inhibited cell proliferation. H460 and H1299 cells were treated with 6-gingerol (100 µM) for 24 h. (**A**) The cell cycle distribution of 6-gingerol-treated cells was assessed by using flow cytometry, and (**B**) the percentage of cells in each phase of the cell cycle was determined. (**C**,**D**) mRNA expression of the cell cycle arrest-related genes p27 and cyclin D1 was detected by using RT–PCR. (**E**) Confocal images of fragmented DNA in cells treated with 6-gingerol determined by using the TUNEL assay are shown. The data are presented as the mean ± SEM. * *p* < 0.05, compared to untreated cells.

Cell cycle arrest plays a crucial role in complex processes for cell survival or cell death [31]. The most common cancer phenotype observed has a rapid proliferation rate. Thus, we investigated whether 6-gingerol would affect the viability of several LC cell lines. H460, A549, and H1299 cells were treated with various concentrations of 6-gingerol for 24 h. The effect of 6-gingerol on the cell cycle progression of H460, A549, and H1299 cells was assessed using flow cytometry to determine whether 6-gingerol inhibits cell growth by changing cell cycle progression. The results showed that 6-gingerol at a high concentration induced G1-phase cell cycle arrest in H460, A549, and H1299 cells (Figure 3A,B). We further investigated the expression levels of several important cell cycle-related genes, such as cyclin D and p21, using RT–PCR to confirm cell cycle arrest. The results demonstrated that 6-gingerol treatment increased the levels of p21 mRNA expression and suppressed cyclin D mRNA expression in H460, A549, and H1299 cells (Figure 3C,D). These results suggest that 6-gingerol promoted cell death by inhibiting the G1-phase transition in the LC cell lines. In addition, we further investigated whether 6-gingerol can induce DNA fragmentation using a terminal deoxynucleotidyl transferase-mediated dUTP nick-end labeling (TUNEL) assay and IF staining.

The number of TUNEL-positive cells was significantly increased compared with that of the control. This result suggests that the DNA in 6-gingerol-treated cells was fragmented, suppressing cell division and proliferation (Figure 3E).

### 3.3. 6-Gingerol Regulates LC Metastasis via the HIF-1α/HSP 90 Pathway

**Figure 4:**
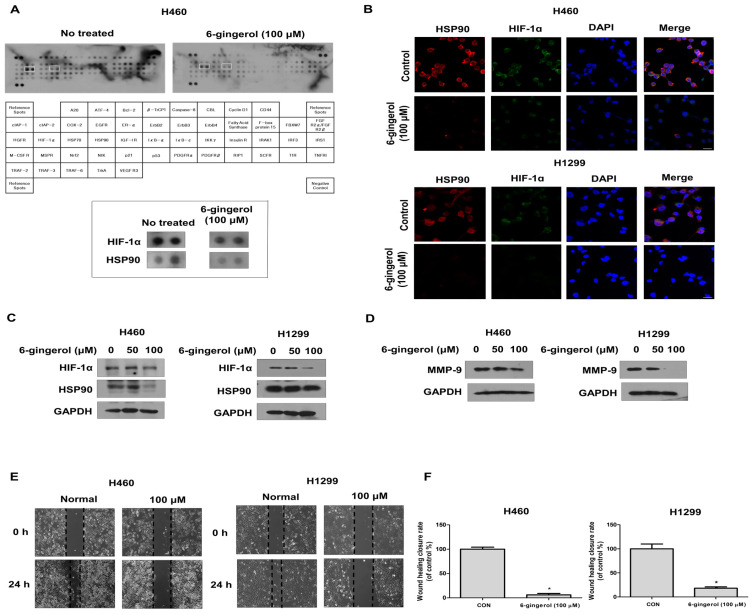
6-Gingerol treatment inhibited metastasis by suppressing the HIF1α/HSP90 pathway under normoxia. H460 and H1299 cells were treated with 6-gingerol (100 µM) for 24 h. (**A**) Whole-cell lysates were analyzed with an antibody array kit. (**B**) Whole-cell lysates were analyzed by Western blotting with anti-HIF-1α, anti-HSP90, and anti-GAPDH antibodies. (**C**) Confocal images demonstrating the subcellular localization of HIF-1α (green) and the HSP90 (red) complex after treatment with 6-gingerol are shown. Nuclei were stained with DAPI (blue) (**D**) The migration of 6-gingerol-treated cells was assessed by using a wound healing assay. (**E**,**F**) Expression of the metastasis-related proteins MMP-9 was detected by using Western blotting. The data are presented as the mean ± SEM. * *p* < 0.05, compared to untreated cells.

To uncover the underlying molecular mechanisms in human LC cells, we first used an antibody array kit. We treated LC cells with 6-gingerol (100 µM) and performed the array. We identified HIF-1α/HSP90 as the major pathway decreased in the 6-gingerol-treated cells (Figure 4A). HIF-1α is a critical oncoprotein that promotes tumor growth involved in angiogenesis and metastasis [26,32]. Previous studies have shown that it is necessary for HSP90 to interact with HIF-1α as a molecular chaperone, which prevents the degradation of HIF-1α transcriptional activity under normoxic conditions [33]. Thus, we studied whether 6-gingerol treatment downregulates HIF-1α/Hsp90 expression levels in human LC cells. We investigated the protein levels of HIF-1α and HSP90 by Western blotting and IF staining. The protein expression levels of HIF-1α and HSP 90 were remarkably downregulated in H460 and H1299 cells, as measured by Western blotting (Figure 4B,C). Additionally, the IF staining results showed that the cytosolic accumulation of HIF-1α was disrupted by 6-gingerol treatment. Taken together, these results indicate that the HIF-1α/HSP90 pathway plays a critical role in inhibiting cancer cell progression.

In addition, we further examined the effect of 6-gingerol on cell migration using Western blotting and wound healing assays, and the results showed that 6-gingerol treatment inhibited cell migration. The results of Western blot analysis showed the inhibition of MMP-9 proteins in a dose-dependent manner in 6-gingerol-treated H460 and H1299 cells (Figure 4D). Furthermore, the scratch wound assay demonstrated that 6-gingerol treatment significantly decreased the rate of cell wound closure compared with that in the control (Figure 4E,F). These data demonstrated that 6-gingerol can inhibit cell migration during normoxia by suppressing the HIF-1α/HSP90 pathway in LC cells.

### 3.4. CoCl_2_ Induces Hypoxic Conditions in Human LC

**Figure 5:**
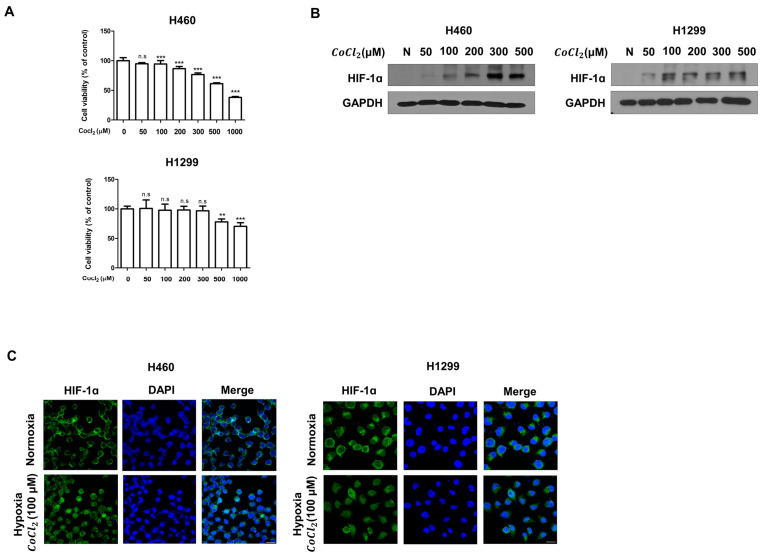
Cobalt chloride (${\mathrm{CoCl}}_{2}$), a chemical agent, induces HIF-1α. H460 and H1299 cells were treated with ${\mathrm{CoCl}}_{2}$ for 24 h. (**A**) H460 and H1299 cells were treated with different concentrations of ${\mathrm{CoCl}}_{2}$ for 24 h. Cell viability was measured by the MTT assay. (**B**) Whole-cell lysates were analyzed by Western blotting with anti-HIF-1α and anti-GAPDH antibodies. (**C**) Confocal images demonstrating the subcellular localization of HIF-1α (green) in cells treated with ${\mathrm{CoCl}}_{2}$ are shown. Nuclei were stained with DAPI (blue). The data are presented as the mean ± SEM, n.s no significance, ** *p* < 0.01, and *** *p* < 0.001 compared to untreated cells.

CoCl_2_, an agent that mimics hypoxia, enhanced by stabilizing HIF-1α [34,35,36]. Thus, we determined the protein levels of HIF-1α LC cells treated with ${\mathrm{CoCl}}_{2}$ under hypoxia. Thus, we investigated whether ${\mathrm{CoCl}}_{2\;}$ influences the viability of LC cells. H460 and H1299 cells were treated with various concentrations of ${\mathrm{CoCl}}_{2}$ for 24 h. Then, cell viability was measured by the MTT assay. The results showed no significant difference in cell survival between the cells with or without ${\mathrm{CoCl}}_{2}$ treatment. However, we found that CoCl_2_ at a high concentration induced cell death (Figure 5A). In addition, we investigated the level of HIF-1α expression in LC cells cultured for 24 h with ${\mathrm{CoCl}}_{2}$ at different concentrations by Western blotting and IF staining (Figure 5B). The Western blotting results showed that ${\mathrm{CoCl}}_{2}$ induced the upregulation of HIF-1α protein expression under normoxia in a dose-dependent manner. Furthermore, the IF staining results showed that the nuclear translocation of HIF-1α was significantly increased under hypoxia (Figure 5C). Taken together, our results show that ${\mathrm{CoCl}}_{2}$ treatment plays an important role in inducing hypoxia, which significantly increases the nuclear translocation and accumulation of HIF-1α.

### 3.5. 6-Gingerol May Inhibit Hypoxia-Induced Expression of HIF-1α/HSP90 and Metastasis in Human LC Cells

In response to a reduced oxygen level, activation of HIF-1α regulates the transcription of a set of hypoxia-response proteins, including those involved in processes such as invasion and tumor cell migration, which are related to tumor progression [37]. Clinical studies have demonstrated that hypoxia is a common feature of solid tumors and that increased HIF-1α is associated with increased patient mortality [38,39]. HSP90 is a major partner of different transcription factors, and hypoxia induces the expression of a transcription-activated form of HIF-1α [40]. Thus, we investigated whether 6-gingerol treatment would downregulate cancer cell growth and target genes through the HIF-1α/HSP90 complex pathway under hypoxia.

**Figure 6:**
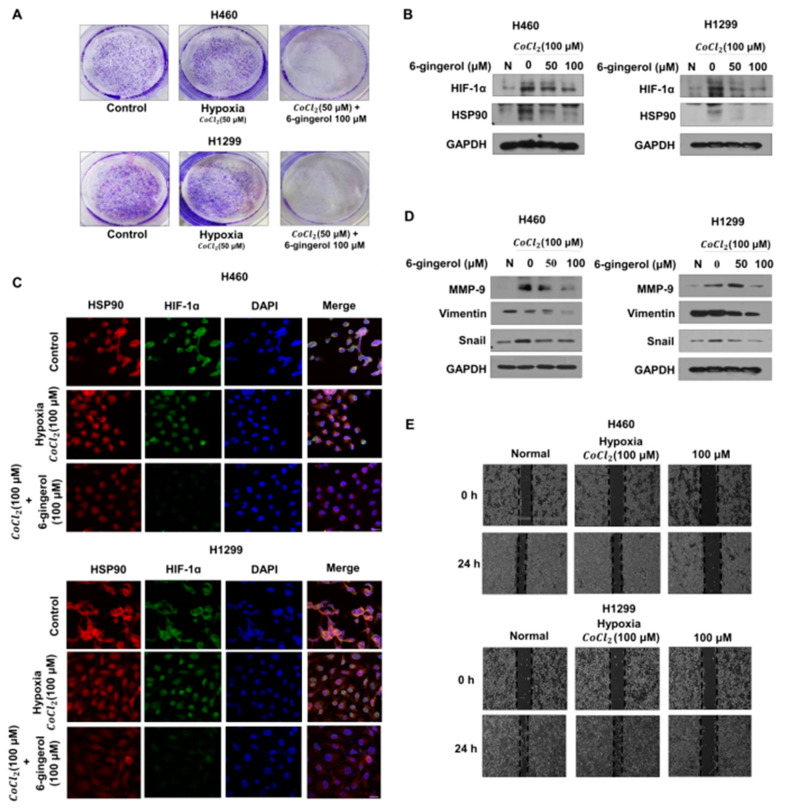
6-Gingerol treatment inhibited metastasis by disrupting HIF-1α stabilization by decreasing the protein level of HSP90 under hypoxia. Pretreatment with ${\mathrm{CoCl}}_{2}$ for 4 h was followed by 6-gingerol treatment. (**A**) H460, A549 and H1299 cells were exposed to 6-gingerol for 7 days. The effect on cell proliferation under hypoxia was assessed by using a colony formation assay. (**B**) Whole-cell lysates were analyzed by Western blotting with anti-HIF-1α and anti-GAPDH antibodies. (**C**) Confocal images demonstrating the subcellular localization of HIF-1α (green) and the HSP90 (red) complex after treatment with 6-gingerol under hypoxia are shown. Nuclei were stained with DAPI (blue). (**D**) Whole-cell lysates were analyzed by Western blotting with anti-HIF-1α and anti-GAPDH antibodies. (**E**) The migration of 6-gingerol-treated cells under hypoxia was assessed by using a wound healing assay. The data are presented as the mean ± SEM. compared to untreated cells.

To study the effect on proliferative ability, we performed a colony formation assay under hypoxic conditions. The results demonstrated a significantly reduced number of colonies and cell colony size compared with those of the control group (Figure 6A).

Next, we studied the mechanism of the effects of 6-gingerol under hypoxic conditions and performed Western blotting and IF staining. We found by Western blotting that 6-gingerol decreased HIF-1α/HSP90 protein expression levels (Figure 6B). Moreover, the IF staining results showed that the nuclear accumulation of HIF-1α/HSP90 was disrupted by 6-gingerol treatment, suggesting that 6-gingerol treatment interferes with the ability of HIF-1α to bind DNA by modulating the transcription-suppressing activity of HIF-1α/HSP90 (Figure 6C). Moreover, to determine whether 6-gingerol treatment is involved in cancer metastasis, we confirmed EMT markers and cell migration in hypoxia-induced cells. As indicated by the number of migrated cells, hypoxia increased the migration of cells compared to that under normoxia, while 6-gingerol inhibited migration during hypoxia in the human LC cell lines. To further confirm the results of Western blot analysis, we showed inhibition of the MMP-9, vimentin and snail proteins in 6-gingerol-treated LC cells (Figure 6D,E). Taken together, our results suggest that 6-gingerol treatment downregulates protein levels in metastasis-activated LC cells by blocking hypoxia-induced transcriptional activity.

### 3.6. 6-Gingerol Treatment Suppressed Cell Growth In Vivo

**Figure 7:**
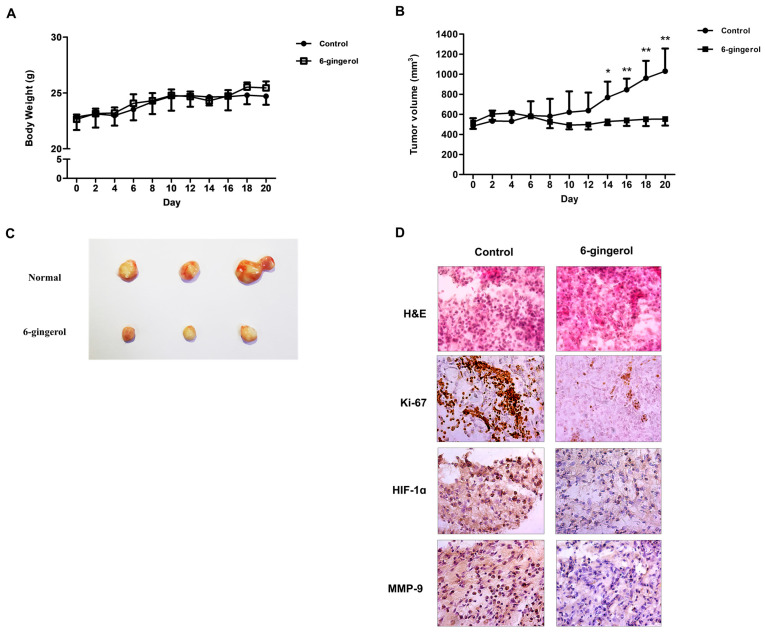
6-Gingerol suppressed LC cell growth in mice. BALB/c nude mice were subcutaneously injected with H460 cells. (**A**) The tumor growth rate and (**B**) mouse body weight are shown. (**C**) The tumor images; (**D**) IHC staining of Ki-67, MMP-9, and HIF-1α was carried out. The data are presented as the mean ± SEM. * *p* < 0.05, ** *p* < 0.01, and compared to untreated cells.

To further confirm the cell growth-inhibitory potential of 6-gingerol, H460 cells were subcutaneously injected into nude mice. The size of xenograft tumors from the 6-gingerol-treated mice indicated a lower growth rate compared to that in the control mice (Figure 7A). However, we found no obvious body weight loss in the two groups (Figure 7B,C). Subsequently, we investigated the levels of Ki-67, HIF-1α and MMP-9 using immunochemical staining. These results showed that the protein levels of Ki-67, HIF-1α and MMP-9 were decreased in the 6-gingerol-treated group (Figure 7D). Together, the above results showed that 6-gingerol inhibited LC proliferation and tumor growth in vivo.

## 4. Discussion

LC is the leading global cause of cancer-related death. Despite the emergence of various treatments, the prognosis of LC is still not satisfactory, as many problems related to LC treatments, including resistance to chemical drugs and unexpected side effects, have been discovered [41,42].

Recent studies have demonstrated hope for disease management by natural dietary substances, also known as phytochemicals, which are more effective and without toxic effects. In particular, *Zingiber officinale Roscoe* is has been commonly used in food and pharmaceutical products. At present, 6-gingerol used in this study is one of the phytochemical agents in *Zingiber officinale Roscoe*. Preclinical studies demonstrated that 6-gingerol has an anti-cancer activity by inhibiting various pathway. In addition to, several clinical studies have suggested beneficial effects of ginger for the treatment of anti-cancer. In fact, for the duration of our in vivo, we did not detect toxic effects, even at molar concentration of 6-gingerol. Ginger extracts are generally regarded as safe. Based on the data presented here, we would argue that future studies should endeavor to look specifically at the effect 6-gingerol could thus provide a useful component of dietary or pharmacological treatment for further drug-synthesis to develop novel and potent clinical candidates. Taken together, these findings suggest that 6-gingerol could be developed as one of the effective chemo preventive or chemotherapeutic agents in many cancers.

In this study, we investigated anticancer of 6-gingerol against LC. Our results demonstrate that 6-gingerol treatment induced cell cycle arrest by increasing the RNA expression of p21 and deactivating cyclin D1. Cell cycle arrest is known to be the main mechanism that controls the proliferation of cancer cells and cancer progression. Thus, 6-gingerol treatment suppressed the proliferation of LC cells.

It has been widely reported that low oxygenation, which arises as a consequence of imbalance between the oxygen supply and oxygen consumption, is a hallmark of solid tumors [43]. HIF-1α is a crucial regulator of oxygen homeostasis, and its transcription is modulated by Hsp90 expression [44]. As a chaperone, Hsp90 is the most abundant protein in the cytosol, and HSP90 interacts with HIF-1α and inhibits HIF-1α ubiquitination, ultimately leading to HIF-1α stabilization, which is correlated with tumorigenesis and progression [45]. In the present study, our results showed that 6-gingerol treatment induced cell death by decreasing the expression levels of HIF-1α and HSP90 in the cytosol under normoxia. These results suggest that the anticancer effects of 6-gingerol treatment on LC cells were mediated by the HIF-1α/HSP90 signaling pathway.

Although the interaction of HIF-1α with Hsp90 has been previously investigated, the physiological importance of the role of Hsp90 in the HIF-1α mechanism has remained elusive. A previous study demonstrated that the stability of the HIF-1α protein is dependent on the oxygen concentration [46]. Our results confirmed that HSP90 and HIF-1α protein expression under normoxia was higher in ${\mathrm{CoCl}}_{2}$-treated cancer cells than in untreated cells. Furthermore, under normoxia, HIF-1α is degraded by the proteasome in an oxygen-dependent manner. In contrast, HIF-1α degradation can also be mediated by HSP90in an oxygen-independent manner [47]. HIF-1α stabilization is linked to the protective function of HSP90 against the proteasomal degradation of HIF-1α through its ATPase activity [40,48]. However, Hsp90 was recently reported to retain the ability to associate with HIF-1α under hypoxia. According to a previous report, an HSP90 inhibitor reduced the hypoxia-mediated activity of HIF-1α [45]. Taken together, these results suggest that Hsp90 associates with HIF-1α in normoxia and is necessary to obtain an active form of HIF-1α under hypoxia. Our results show that 6-gingerol treatment induced cell death by decreasing the expression levels of HIF-1α and HSP90 in the cytoplasm and nucleus under hypoxic conditions. Furthermore, our results suggest that HSP90 plays a pivotal role as a master regulator of HIF-1α protein stability under both normoxia and hypoxia. However, the role of HSP90 in regulating the nuclear translocation of HIF-1α under hypoxia remains unclear, and further studies are required to confirm whether HIF-1α is a target of HSP90.

In addition, as it is a transcription factor, HIF-1α is correlated with dozens of target genes involved in processes such as proliferation, metastasis and angiogenesis. Our results demonstrated that 6-gingerol treatment inhibited HIF-1α target genes, including MMP-9, vimentin and snail, which are correlated with tumor metastasis. Thus, the findings bring new insight into the metastatic pathway in LC cell lines.

Taken together, our findings show that 6-gingerol negatively regulates hypoxia-induced metastasis by inhibiting the HIF-1α/HSP90 signaling pathway both in vitro and in vivo. Furthermore, our results suggest that 6-gingerol is a novel therapeutic target for inhibiting cancer metastasis in LC. However, our results do not provide a good understanding of the molecular mechanism underlying the modulation of HIF1 stability under different conditions. Further research on this topic is needed to understand the mechanisms of potential therapeutic targets in LC cells.

## Figures and Tables

**Table 1:** Sequences of primers used for RT–PCR.

Type	Primer Name	Sequences	
Human	p27	Forward	5′-TCA AAC GTG CGA GTG TCT AAC-3′
		Reverse	5′-AAT GCG TGT CCT CAG AGT TAG-3′
Human	Cyclin D1	Forward	5′-CTG GCC ATG AAC TAC CTG GA-3′
		Reverse	5′-GTC ACA CTT GAT CAC TCT GG-3′
Human	GAPDH	Forward	5′-CGT CTT CAC CAC CAT GGA GA-3′
		Reverse	5′-CGG CCA TCA CGC CAC AGT TT-3′

## Data Availability

All data and materials are contained and described within the manuscript.
